# Development and validation of scFv-conjugated affinity silk protein for specific detection of carcinoembryonic antigen

**DOI:** 10.1038/s41598-017-16277-6

**Published:** 2017-11-22

**Authors:** Mitsuru Sato, Hiroshi Kitani, Katsura Kojima

**Affiliations:** 1 Animal Bioregulation Unit, Division of Animal Sciences, Institute of Agrobiological Sciences, National Agriculture and Food Research Organization, 1-2 Owashi, Tsukuba, Ibaraki 305-8634 Japan; 2 Silk Materials Research Unit, Division of Biotechnology, Institute of Agrobiological Sciences, National Agriculture and Food Research Organization, 1-2 Owashi, Tsukuba, Ibaraki 305-8634 Japan

## Abstract

The production costs for monoclonal antibodies (MAbs) utilized in medical diagnostic kits are inevitably high because the MAbs are mostly obtained from hybridoma cell culture. Here, we report the development and validation of a novel affinity silk protein produced by transgenic silkworm technology as a possible alternative diagnostic tool for cancers. We generated a transgenic silkworm expressing a cDNA construct containing fibroin L-chain fused to a single-chain variable fragment (scFv) derived from a MAb against carcinoembryonic antigen (CEA). The transgenic cocoons were dissolved in aqueous lithium bromide solution, applied to 96-well plates, and analysed by enzyme-linked immunosorbent assay. The scFv-conjugated affinity silk protein specifically recognized CEA as well as the parental MAb. The binding activity was retained after several months of storage in coated plates or concentrated solution. Thus, the scFv-conjugated affinity silk protein provides a potentially useful alternative to conventional MAbs in medical diagnostic kits.

## Introduction

Monoclonal antibodies (MAbs) are utilized in various medical applications, such as the diagnosis of infectious diseases or cancers, in either 96-well plates or specifically designed devices. However, the overall production cost is inevitably high because MAbs are mostly obtained from hybridoma cells, which require expensive foetal bovine serum and equipped cell culture facilities. In addition, many laborious processes are required, such as purification, modification, and integration of MAbs to make the specific detection device. Therefore, the development of an alternative strategy that could save production cost and time is important for the industrial utilization of these affinity reagents.

Recent advances in transgenic silkworm technology have demonstrated that recombinant proteins can be produced in the silk glands, either independently from silk proteins^1,2^ or fused with fibroin proteins^3–6^. In the latter case, recombinant proteins maintain their structural and functional property in a conjugated form with the silk fibroin molecules. To evaluate whether transgenic silk fibroins can be used as a novel affinity material, we previously produced a transgenic silkworm strain that spins silk protein containing the fibroin L-chain (FibL) fused to the single-chain variable fragment (scFv)^7,8^. In that work, the scFv construct (composed of V_H_ and V_L_ domains) was originated from a MAb against Wiskott-Aldrich syndrome protein (WASP), which acts as an adaptor molecule in mammalian immune cells^9–12^. The transgenic cocoons were dissolved in aqueous lithium bromide (LiBr) solution, then the silk solution was dialysed, concentrated, freeze-dried, and processed into powder. This scFv-conjugated silk powder specifically immunoprecipitated its target protein, WASP^13^. In addition, we processed cocoons expressing anti-WASP scFv-conjugated fibroin protein into a thin film by dissolving it in LiBr solution and drying it in 96-well plates to demonstrate the specific detection of WASP by enzyme-linked immunosorbent assay (ELISA)^14^. These results clearly suggested that scFv-conjugated affinity silk protein, in powder or film form, can specifically recognize the target protein and provide useful alternatives for affinity purification or immunodetection of the antigen, respectively.

In the present work, we produced a transgenic silkworm strain that spins silk protein containing FibL fused to scFv derived from a MAb against carcinoembryonic antigen (CEA). CEA is a member of a family of cell surface glycoproteins over-produced in a variety of malignancies^15^. Although CEA is most commonly associated with colorectal cancer, it can also be elevated in other malignancies, such as breast, liver, stomach, and pancreas cancer, suggesting that CEA is most widely used as a tumour-associated serum biomarker^16,17^. Cocoons containing anti-CEA scFv-conjugated affinity silk protein were dissolved and the specific binding activity for the target antigen evaluated by ELISA. Our present study demonstrates that scFv-conjugated affinity silk protein could be potentially used as an alternative to conventional affinity reagents for the diagnosis of human cancer.

## Results

### Construction of scFvs, expression in mouse T cells, and CEA binding assay

**Figure 1:**
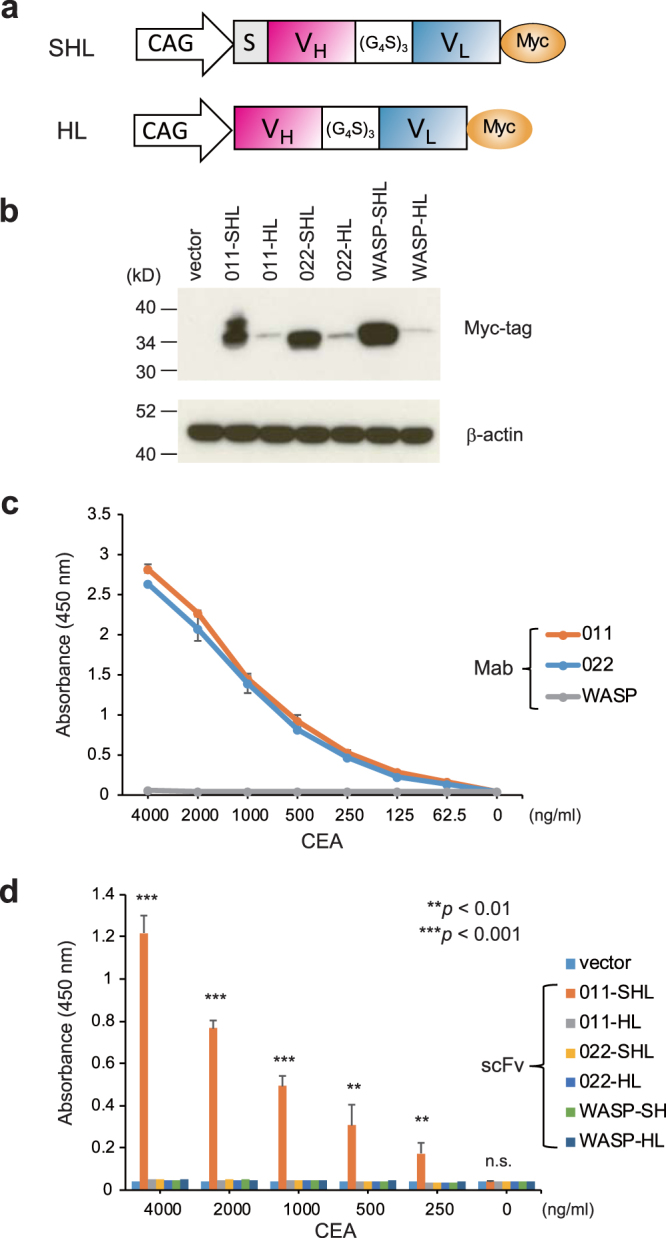
Anti-CEA scFv constructs and their expression in mouse T cells and CEA binding assay. (**a**) Schematic representation of anti-CEA SHL and HL constructs. The CAG promoter, leader signal sequence (S), V_H_ and V_L_ regions of anti-CEA MAb, flexible peptide linker [(G_4_S)_3_], and Myc-tagged sequence are shown. (**b**) Western blot of anti-CEA 011-SHL, 011-HL, 022-SHL, 022-HL, anti-WASP-SHL, and-HL scFvs in transfected DO11-10 T cells. The immunoblots were probed with anti-Myc-tag polyclonal antibody or anti-β-actin MAb. Full-length gels/blots are presented in Supplementary Figure S2. (**c**) Binding assay of parental anti-CEA MAbs (011 and 022) and a control anti-WASP MAb by ELISA. (**d**) Binding assay of anti-CEA or anti-WASP scFvs by ELISA. T cell lysates transfected with DNA encoding anti-CEA and anti-WASP scFvs were used. The indicated amount of CEA protein was added to each well. Values are presented as mean ± standard error of duplicate reactions and are representative of three independent experiments. ** *P* < 0.01; *** *P* < 0.001; n.s., not significant.

To generate scFv from hybridomas, four-step PCR was performed with appropriate primers for amplification and assembly of the V_H_ and V_L_ regions (Figure S1 and Table S1). We constructed two types of scFvs derived from two clones of anti-CEA monoclonal antibodies with or without their V_H_ leader sequences: 011-SHL, 011-HL, 022-SHL, and 022-HL. (Fig. 1a). The constructs were transiently transfected into DO11-10 murine T cells^18^. Western blot with anti-Myc-tag antibody showed efficient expression of anti-CEA-scFv 011-SHL and 022-SHL containing their V_H_ leader sequences in mouse T cells (Fig. 1b, upper panel), but not the 011-HL and 022-HL constructs without the leader sequences. These results suggest that the efficiency of scFv construct expression is highly dependent on the presence of V_H_ leader sequences in mouse T cells, as demonstrated previously in anti-WASP scFvs^11^. The amount of protein loaded in each lane was equal, as confirmed by Western blotting with anti-β-actin antibody (Fig. 1b, lower panel).

To assess the binding activity of anti-CEA scFvs to the target CEA protein, the lysates from scFv gene-transfected T cells were coated on 96-well plates and analysed by ELISA. As a positive control, both of the parental MAbs (clones 011 and 022) exhibited similar specific binding to CEA (Fig. 1c), but a negative control anti-WASP MAb did not. Regarding anti-CEA-scFvs, only 011-SHL-coated wells had a specific reaction in an antigen concentration-dependent manner (Fig. 1d). The other three constructs (011-HL, 022-SHL, and 022-HL) did not react to CEA. The 011-HL and 022-HL constructs were hardly expressed in gene-transfected T cells, but the 022-SHL construct had a similar expression level as 011-SHL (Fig. 1b). These results suggest that antigen-specific binding of scFv is not simply determined by its expression level. Conversely, the structural property of antigen binding by the scFv should be maintained in mouse T cells.

**Figure 2:**
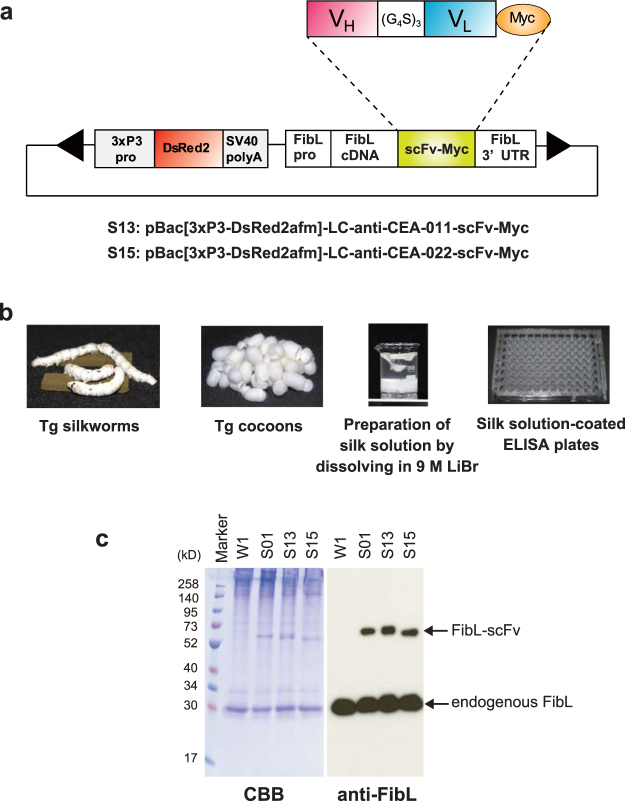
Construction of plasmids and production of affinity silk protein from transgenic silkworms. (**a**) Schematic representation of the DNA plasmid for S13 and S15 transgenic silkworm strains. The plasmid contains expression units for the selection marker and recombinant protein between the *piggyBac* repeated terminal sequences (arrowheads). The 3xP3 promoter (3xP3pro), DsRed2 gene, SV40 polyA signal sequence, fibroin L-chain promoter (FibLpro), cDNA of fibroin L-chain (FibL cDNA), cDNA of anti-CEA-scFv fused with a Myc-tag sequence (scFv-Myc), and fibroin L-chain 3′-untranslated region (FibL-3′UTR) are shown. (**b**) Schematic illustration for preparing silk solution-coated plates. Cocoons produced by transgenic silkworms were dissolved in 9 M LiBr solution, and then coated on 96-well plates. (**c**) SDS-PAGE and Western blot analysis of the expression of transgenes FibL-anti-CEA-scFv-Myc (S13 and S15) and control FibL-anti-WASP-scFv-Myc (S01) in the silk solution. Silk solutions derived from wild-type (W1), S01, S13, and S15 strains were analysed by SDS-PAGE and Coomassie brilliant blue staining. Immunoblots were probed with anti-FibL polyclonal antibody.

Western blot analysis with anti-Myc-tag antibody detected 011-SHL as two bands (Fig. 1b, upper panel). The upper band was derived from the intact scFv conjugated to the leader sequence, and the lower band was derived from the truncated scFv with the leader sequence cleaved at the N-terminal of the V_H_ region. This truncated 011-SHL scFv had a similar molecular weight as the 011-HL construct, which did not contain the leader sequence (Fig. 1b, upper panel). Most of the 022-SHL and WASP-SHL constructs were cleaved and their leader sequence lost in the transfected T cells. Therefore, we used leader-less constructs (011-HL and 022-HL) for the generation of transgenic silkworms. In addition, the fibroin L-chain derived leader sequence was contained in the N-terminus of FibL cDNA construct (Fig. 2a).

### Generation of transgenic silkworms which produce scFv-conjugated affinity silk protein in cocoon shells

We generated two transgenic silkworm strains, S13 and S15, that spun silk containing FibL fused to anti-CEA-011-scFv and 022-scFv, respectively (Fig. 2a). Wild-type w1-pnd (W1) and transgenic strain S01^13,14^, which produces FibL-anti-WASP-scFv, were used as controls. The cocoons were cut into small pieces and dissolved in 9 M LiBr solution (Fig. 2b). Expression of the transgenes composed of the S13 and S15 constructs or control S01 construct was verified in each silk solution by sodium dodecyl sulphate polyacrylamide gel electrophoresis (SDS-PAGE), followed by Coomassie brilliant blue (CBB) staining and immunoblot analysis using anti-FibL antibody (Fig. 2c). The levels of scFv-conjugated FibL expression were estimated to be approximately 10% of endogenous FibL by densitometric analysis, which is comparable to the average expression levels in the previous transgenic silk experiments^3–5,13^. These results demonstrate that anti-CEA-011-scFv and 022-scFv constructs fused with FibL are sufficiently expressed as fusion proteins in silk fibres in the transgenic cocoon shells.

### ScFv-conjugated affinity silk solution detects the target CEA protein in ELISA

**Figure 3:**
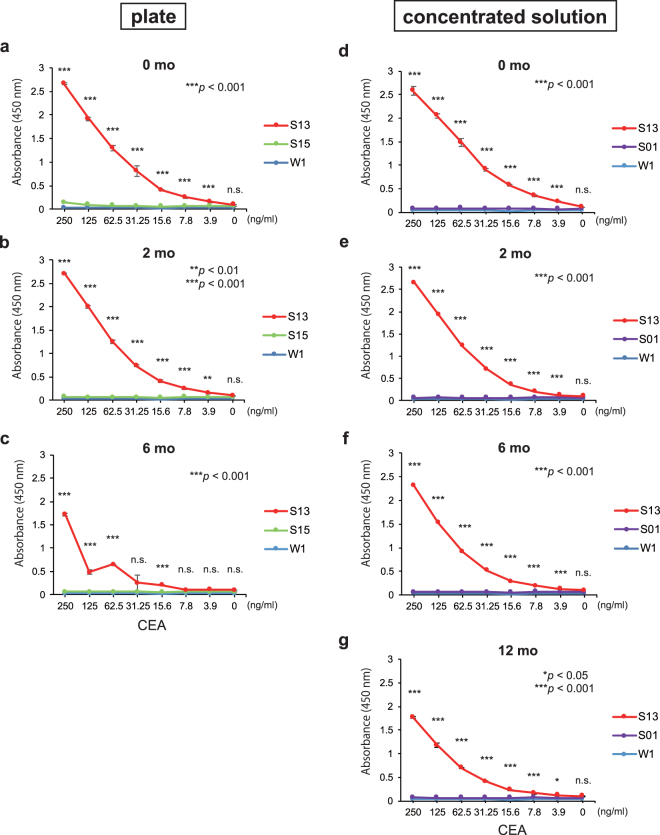
ScFv-conjugated silk solution specifically detected CEA protein in ELISA. (**a**–**c**) Freshly prepared plates (0 months) or after storage (2 or 6 months) in plates at 4 °C. (**d**–**g**) In the other assay, W1, S01, or S13 cocoon shells were dissolved in aqueous LiBr solution and stored in liquid form at 4 °C for 2, 6, or 12 months and compared to freshly prepared solution (0 months) for specific binding to CEA. The indicated amount of CEA was applied to each silk solution-coated well. Values are presented as mean ± standard error of duplicate reactions and are representative of three independent experiments. * *P* < 0.05; ** *P* < 0.01; *** *P* < 0.001; n.s., not significant.

The specific binding of scFv-conjugated affinity silk solution for the target CEA protein was verified by ELISA, either in freshly prepared plates or after storage for 2 and 6 months at 4 °C. In the freshly prepared plates, the intensity of absorbance of S13 silk solution (anti-CEA-011-scFv)-coated wells increased as the concentration of CEA increased (Fig. 3a). However, S15 silk solution (anti-CEA-022-scFv)-coated wells did not react to CEA protein, despite the similar levels of expression in the cocoon shells (Fig. 2c). This may be explained by the inability of anti-CEA-022-scFv to retain proper folding, which is critical for recognition of the antigen in scFv format, as demonstrated in mouse T cells (Fig. 1d). As a negative control, the wild-type W1 silk solution-coated wells did not exhibit a positive reaction (Fig. 3a). The sensitivity of S13 silk solution-coated plates did not change after storage for 2 months at 4 °C (Fig. 3b), and remarkably decreased after 6 months (Fig. 3c). These results suggest that the binding activity of S13 silk solution-coated plates can be retained for at least 2 months at 4 °C. Taken together, anti-CEA-011-scFv fused to FibL in S13 transgenic silkworm preserves the appropriate folding which is essential for antigen binding during dissolution, coating, washing, and preservation steps.

To investigate whether the scFv-conjugated silk solution can be stably preserved for a longer time, W1, S01, and S13 cocoon shells were dissolved in aqueous LiBr solution and stored in liquid form at 4 °C for the indicated times. Just before the analysis, the stock silk solutions (80 mg/mL in 9 M LiBr) were appropriately diluted, applied to 96-well plates, and the binding activity determined by ELISA. The absorbance intensity of freshly prepared S13 silk solution increased as the concentration of CEA increased (Fig. 3d). The sensitivity of S13 silk solution barely decreased after storage for 2 months (Fig. 3e) and only slightly decreased after 6 months (Fig. 3f). Even after 12 months of storage, the specific reaction was still observed, though at half the level of freshly prepared S13 silk solution (Fig. 3g). As negative controls, W1 and S01 silk solution did not show a non-specific reaction in these assays regardless of the storage period. These results suggest that the binding activity of S13 silk solution containing anti-CEA-011-scFv-conjugated FibL can be stably preserved for at least 6 months when dissolved in 9 M LiBr solution and kept at 4 °C. In our study, scFv-conjugated silk solution was prepared from 300 mg cocoon shells (~2 to 3 shells), and this amount of protein is usually sufficient to coat nearly 100 96-well plates. The production cost for the transgenic silkworm cocoons varies from farmer production to laboratory production, but can be estimated as roughly US$1 per cocoon. Thus, the production cost for the ELISA plates coated with scFv-conjugated silk solution can be substantially lower than those conventionally coated with monoclonal antibodies.

### Binding specificity of scFv-conjugated silk solution

**Figure 4:**
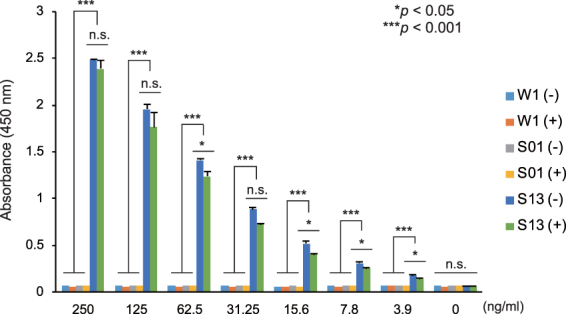
Binding specificity of scFv-conjugated silk solution. Specific reaction was quantified by ELISA using 96-well plates coated with silk solution prepared from W1, S01, or S13 strains. The indicated amount of CEA was applied to each silk solution-coated well and incubated in the presence (+) or absence (−) of 10% mouse serum. Values are presented as mean ± standard error of duplicate reactions and are representative of three independent experiments. * *P* < 0.05; *** *P* < 0.001; n.s., not significant.

To evaluate the binding specificity for the target protein, W1, S01, and S13 silk solutions were prepared and applied to 96-well plates. After blocking each well, CEA protein was incubated in the presence or absence of 10% normal mouse serum and the antigen-specific binding determined by ELISA. If the anti-CEA scFv-conjugated S13 silk protein non-specifically reacts to normal mouse serum proteins, the specific reaction will decrease. However, the specific binding of S13 silk solution to CEA was not severely influenced by the presence of normal mouse serum (Fig. 4). In addition, non-specific binding to normal mouse serum proteins was not observed in any of the silk solution-coated wells (Fig. 4), suggesting that S13 scFv-conjugated and control silk solution do not cross-react with non-related mouse serum proteins. Therefore, the scFv-conjugated solution can be used for specific detection of the target CEA protein in quantitative ELISA. The level of CEA protein in healthy persons is less than 5 ng/mL, and the CEA > 10 ng/mL is diagnosed as possible gastrointestinal cancer^19^. The ELISA plates coated with anti-CEA scFv-conjugated silk solution had sufficient sensitivity to detect these CEA protein levels (Fig. 4). Taken together, silk solution prepared from transgenic silk cocoon shells containing scFv fused to the FibL would provide a useful alternative for manufacturing novel immunodetection systems at lower cost and labour while retaining sufficient affinity and specificity for the target protein after several months of storage.

## Discussion

CEA is a well-known biomarker used for screening gastrointestinal cancers and an important prognostic marker for cancer treatment, recurrence, and metastasis. In the present study, we produced a transgenic silkworm strain that spun silk fibres containing anti-CEA-scFv-conjugated FibL. The dissolved silk protein derived from transgenic cocoon shells sufficiently detected the target CEA in the ELISA system, and its binding activity was stably preserved in solution for at least 6 months. These observations clearly suggest that scFv-conjugated silk protein would provide a novel alternative to MAbs for constructing various medical diagnostic tools that can be produced at lower cost.

The composition of the anti-CEA-scFv transgenic silk solution is considered to be similar to that of the wild-type cocoon shell: sericin (20% w/w), fibroin H-chain (72.2% w/w), FibL (6.8% w/w), and fibrohexamerin (fhx)/P25 (1% w/w)^20^. The proportion of scFv-conjugated FibL was estimated to be only 1% or less of the total silk proteins in the solution. Nevertheless, these scFv-conjugated FibL were sufficient for specific binding to CEA in ELISA. The activity could be improved by increasing the proportion of scFv-conjugated FibL in the silk solution. However, further processing, such as degumming, which is used for the isolation and purification of fibroin^21^, would destroy the biological activity of scFv-conjugated FibL.

In addition to expression levels, the proper conformation of scFv should be maintained in the conjugated form with FibL. To develop a new affinity silk material that specifically recognizes CEA, we constructed anti-CEA-011 and 022-scFvs derived from two independent MAbs against CEA. When these scFv constructs were transiently transfected into mouse T cells, their expression levels were almost similar (Fig. 1b). However, the efficient CEA binding activity was detected with anti-CEA-011-scFv, but not with anti-CEA-022-scFv, suggesting that anti-CEA-011-scFv can retain the proper folding structure required for antigen binding when antibody fragments are integrated into the scFv format in mouse T cells. Similar observations were made in the two transgenic silkworm strains, S13 and S15. Though the expression level of each FibL-anti-CEA-scFv fusion protein in silk solution was similar, specific binding to CEA was only observed for S13, but not in S15, silk solution. These results suggest that the anti-CEA-011-scFv construct spontaneously acquires its appropriate conformation in the fused form with FibL when silk proteins are produced and secreted into the silk gland lumen. Furthermore, the proper conformation required for antigen binding was retained during the dissolution and coating processes. Given these observations, a critical factor for the success of affinity silk production is the selection of scFv constructs that can preserve the proper conformation for specific binding in the scFv format. This property can be checked in mammalian cells in advance. In our previous work, we used a specific anti-WASP-scFv construct, which was confirmed to bind to WASP protein in mouse T cells^11,22,23^, and successfully produced a transgenic silkworm strain that provides affinity silk powder^13^ and film^14^.

Traditional ELISA systems require the antigen-capturing MAbs, which are mainly produced by hybridomas, followed by isolation and purification of the antibody. In contrast, affinity silk solution requires only one step, which includes dissolving the transgenic cocoon shells in LiBr solution. In addition, this silk solution can be stored stably for at least 6 months at 4 °C. Therefore, the cost of manufacturing ELISA systems with affinity silk protein would be far less than the cost required for traditional methods that depend on monoclonal antibodies. Moreover, the most practical advantage of using silk proteins is that the transgenic cocoon shells can be stored at room temperature for many years without significant loss of their biological activities. For example, anti-WASP-scFv-conjugated S01 affinity silk solution prepared from transgenic cocoon shells stored for nine years at room temperature still specifically detected the target protein in ELISA (Figure S3) at similar levels compared to that from freshly obtained cocoon shells^14^. In addition, the cocoon shells containing fibroin fused with enhanced green fluorescent protein^13^ are able to emit fluorescence after several years of storage at room temperature in our laboratory. With this unique property, affinity silk protein solutions can be prepared from the cocoon shell stocks on demand and integrated into specific medical devices, such as filters or masks, which could capture and remove infectious pathogens or allergens in the air.

Affinity silk protein can be used to modify polystyrene and magnetic microbeads for the purification and detection of the target proteins. Furthermore, recent progress in silk fibroin-derived nanoparticle technology^24,25^ may provide a novel approach in biomedical applications to utilize scFv-conjugated affinity silk. Silk fibroin-derived nanoparticles have been evaluated as a novel drug-delivery platform^26–28^. Thus, affinity silk nanoparticles that specifically recognize a biomarker specific for cancer cells and other disease-associated cells would provide new strategies for therapy without concomitant side effects.

## Methods

### Preparation of monoclonal antibodies

The hybridoma clones producing MAbs against CEA, EB-011 and EB-022, were provided by Nippon Bio-Test Laboratories, Inc. (Saitama, Japan); they were prepared from mice immunized with human CEA isolated from colon carcinoma liver metastatic tissue using a conventional procedure.

### Cloning and construction of anti-CEA scFvs

Total RNA from hybridoma cells was reverse-transcribed using the SMART^TM^ RACE cDNA amplification kit (Clontech, Mountain View, CA). The cDNA fragments for the V_H_ and V_L_ regions containing the N-terminal leader signal sequence were amplified by PCR with appropriate primers, and then mixed and assembled into scFvs by four-step PCR amplification using appropriate primers containing the linker sequence (Supplementary Figure S1 and Table S1). The resulting fragments were digested with *Not*I and cloned into pCAGGS-MCS expression vector^29,30^. The Myc tag (EQKLISEEDL) was inserted into the *Xba*I*/Eco*RI site of all pCAG/anti-CEA scFv constructs. The result was that all anti-CEA scFvs were fused with the Myc tag at the C-terminus. The GenBank/EMBL/DDBJ accession numbers for the sequences of the cDNAs encoding V_H_ and V_L_ containing the N-terminal leader sequences are: 011-V_H_, LC216402; 011-V_L_, LC216403; 022-V_H_, LC216404; 022-V_L_, LC216405.

### Cells and electroporation

The murine T-cell hybridoma DO11-10 and hybridoma cells producing anti-CEA and anti-WASP antibodies were maintained in RPMI1640 medium supplemented with 100 U/mL penicillin, 100 μg/mL streptomycin, 4 mM _L_-glutamine, 10 mM HEPES (all from Life Technologies, Carlsbad, CA, USA), and 10% foetal calf serum (FCS). DO11-10 cells adjusted to a concentration of 5 × 10^6^ cells/400 μL culture medium with 1.25% dimethyl sulfoxide per cuvette were electroporated using a Gene Pulser (Bio-Rad, Hercules, CA, USA) with 40 μg plasmid DNA at 290 V and 960 μF.

### Construction of plasmids for transgenic silkworms

cDNA fragments for anti-CEA scFv-Myc were generated by PCR from pCAG/anti-CEA-011-scFv-Myc and 022-scFv-Myc using sense primer #11 and reverse primer #12 (Supplementary Table S1). These PCR products were digested with *Bam*HI-*Sal*I, inserted between the FibL promoter region through the FibL coding region (FibLpro-FibL) and FibL 3′-untranslated region (FibL-3′-UTR), and the fused DNA fragments of FibLpro-FibL-anti-CEA-scFv-FibL-3′-UTR cloned into the *Asc*I-*Fse*I site of pBac[3XP3-DsRed2afm] vector^4^; these constructs were designated pBac[3XP3-DsRed2afm]-LC-anti-CEA-scFv-011-Myc and pBac[3XP3-DsRed2afm]-LC-anti-CEA-scFv-022-Myc, respectively.

### Generation of transgenic silkworm strains

Transgenic silkworm strains were produced as described elsewhere^31^ except for several modifications. The transgene plasmid and a helper pHA3PIG plasmid vector coding for *piggyBac* transposase^31^ were mixed at a concentration of 0.2 μg/μL each in 5 mM KCl and 0.5 mM phosphate buffer (pH 7.0) and injected into the fertilized eggs of the w1-pnd silkworm according as described previously^13^. Hatched G0 larvae were reared under standard condition^13^. G1 embryos were screened for transgenic individuals with DsRed2 expression 6 to 7 days after oviposition by fluorescent microscopy (MZ16FA, Leica Microsystems, Wetzlar, Germany), and were reared and sib-mated for at least three generations. S13 and S15 strains carried the transgene coding for the FibL fused with anti-CEA-011-scFv-Myc and anti-CEA-022-scFv-Myc, respectively. Control strain S01 carried the transgene coding for the FibL fused with anti-WASP scFv-Myc^13^.

### Preparation of silk solution from cocoon shells

Three hundred milligrams of cocoon shells (2 to 3 shells) were cut into 2 to 3-mm squares and washed in 70% ethanol, and then dissolved in 9 M LiBr and 90 mM Tris-HCl (pH 9.0) according as described previously^14^. The solubilized silk solutions were adjusted to a concentration of 80 mg/mL in 9 M LiBr as a stock solution.

### Immunoblotting

The gene-transfected DO-11.10 cells and silk solutions from W1, S01, S13, and S15 strains were processed for immunoblot analyses according as described previously^14^. After blocking with Blocking One (Nacalai Tesque, Kyoto, Japan) for 1 h at room temperature and incubated with anti-Myc polyclonal antibody (MBL, code no. 562, Nagoya, Japan), anti-β-actin rabbit MAb (Cell Signaling Technology, code no. #4970, Danvers, MA, USA), and anti-FibL polyclonal antibody^14^, followed by horseradish peroxidase conjugated anti-rabbit immunoglobulins (Igs) (Dako, code no. P0399, Glostrup, Denmark), and imaged with Chemi-Lumi One L (Nacalai Tesque).

### ELISA

The gene-transfected DO-11.10 cells were lysed with RIPA buffer (50 mM Tris-HCL pH 7.6, 150 mM NaCl, 1% Nonidet P-40, 0.5% sodium deoxycholate, and protease inhibitor cocktail; Nacalai Tesque) on ice for 1 h. Cell lysates were centrifuged at 10,000 × *g* for 10 min at 4 °C and the supernatants used for ELISA. The stock silk solutions (80 mg/mL in 9 M LiBr) were diluted with 1 mM Tris-HCl (pH 8.0) to a concentration of 0.25 mg/mL. One hundred microlitres of the cell lysate, culture supernatants from hybridoma cells, and diluted silk solutions were applied to 96-well plates and incubated overnight at 4 °C. After three washes with PBS, each well was blocked with ELISA Assay Diluent (BioLegend, San Diego, CA, USA) at room temperature for 1 h. After five washes with PBS and Tween 20, CEA (Abcam, code no. ab742, Cambridge, UK) was added to the wells and incubated at room temperature for 2 h. Binding was detected with anti-CEA polyclonal antibody (Abcam, code no. ab15987), then HRP-conjugated anti-rabbit Igs (Dako), finally by incubation with ELISA POD Substrate TMB solution (Nacalai Tesque). After colour development, the reaction was stopped with 2 N H_2_SO_4_ and the absorbance read at 450 nm using a microplate reader (iMark^TM^ Microplate Reader; Bio-Rad).

### Statistical analysis

Statistical significance was evaluated using GraphPad Prism 6 (GraphPad Software, La Jolla, CA, USA); the Student’s *t*-test for paired samples and one-way ANOVA with Tukey’s test for multiple samples, respectively. *P* values of < 0.05 were considered statistically different.

## Electronic supplementary material

Supplementary Information
