# Supplementary material for: Development and validation of scFv-conjugated affinity silk protein for specific detection of carcinoembryonic antigen
Source: Sci Rep. 2017 Nov 22;7:16077. doi: 10.1038/s41598-017-16277-6 (PMC5700171; doi:10.1038/s41598-017-16277-6)
Supplement: Supplementary file 1 — Supplementary Information [file 41598_2017_16277_MOESM1_ESM.pdf]

[Supplementary information]

Development and validation of scFv-conjugated affinity silk protein for specific detection of carcinoembryonic antigen

Mitsuru Sato[1†*], Hiroshi Kitani[1], and Katsura Kojima[2†]

[1]Animal Bioregulation Unit, Division of Animal Sciences, and [2]Silk Materials Research Unit, Division of Biotechnology, Institute of Agrobiological Sciences, National Agriculture and Food Research Organization, 1-2 Owashi, Tsukuba, Ibaraki 305-8634, Japan

†These authors contributed equally to this work.

*Address Correspondence to: Mitsuru Sato

Animal Bioregulation Unit, Division of Animal Sciences, Institute of Agrobiological Sciences, National Agriculture and Food Research Organization, 1-2 Owashi, Tsukuba, Ibaraki 305-8634, Japan

E-mail: mitsuru.sato@affrc.go.jp

Tel: +81 29 838 6041

Fax: +81 29 838 6028

Supplementary information Figure S1

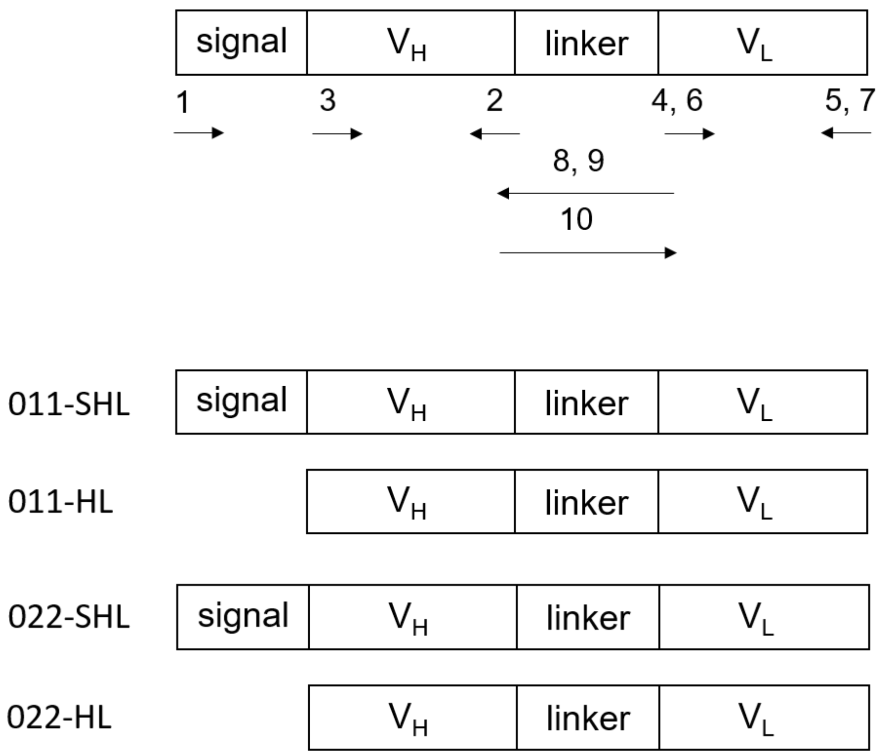

Figure S1. Construction of anti-CEA-scFvs by cloning the variable region of immunoglobulin heavy and light chains from hybridoma cells producing anti-CEA monoclonal antibody. The arrows represent the primers used to amplify the antibody fragments.

The isotype of EB-011 and EB-022 MAb was identified as IgG1 and κ using a mouse MAb isotyping kit, IsoStrip (Roche Diagnostics, Mannheim, Germany). We performed a four-step PCR to generate appropriate cDNA fragments encoding the V_H and V_L regions. Total RNA from hybridoma cells was reverse-transcribed using the SMART^{TM} RACE cDNA Amplification Kit (Clontech, Palo Alto, CA, USA). The cDNA fragments for the V_H and V_L regions containing the leader signal sequence were generated by PCR using isotype-specific primers (heavy chain, IgG1: sense primer 5’-AAGCAGTGGTATCAACGCAGAGTACGCG-3’ and reverse primer 5’-

CCCTGGGCACAATTTTCTTGTCCACC-3’; light chain, κ: sense primer 5’-AAGCAGTGGTATCAACGCAGAGTACGCG-3’ and reverse primer 5’-CCTGTTGAAGCTCTTGACAATGGGTG-3’). The second PCR products for the V_H region were classified into two forms containing the native V_H leader signal sequence at the 5’ end of the V_H fragment (SV_H form) or no V_H leader signal sequence (V_H form). The second PCR amplification was performed with the following primer sets: 011-SV_H and 022-SV_H, sense primer #1 and reverse primer #2; 011-V_H and 022-V_H, sense primer #3 and reverse primer #2; 011-V_L, sense primer #4 and reverse primer #5; 022-V_L, sense primer #6 and reverse primer #7. The third PCR products were amplified using the following primer sets: 011-SV_H-linker, sense primer #1 and reverse primer #8; 011-V_H-linker, sense primer #3 and reverse primer #8; 022-SV_H-linker, sense primer #1 and reverse primer #9; 022-V_H, sense primer #3 and reverse primer #9; linker-011-V_L, sense primer #10 and reverse primer #5; linker-022-V_L, sense primer #10 and reverse primer #7. The third PCR products were mixed in the following combinations: 011-SV_H-linker and linker-011-V_L, 011-V_H-linker and linker-011-V_L, 022-SV_H-linker and linker-022-V_L, 022-V_H-linker and linker-022-V_L and then the fourth PCR amplification was performed with the following primer sets: 011-SHL, sense primer #1 and reverse primer #5; 011-HL, sense primer #3 and reverse primer #5; 022-SHL, sense primer #1 and reverse primer #7; 022-HL, sense primer #3 and reverse primer #7. The fourth PCR products were digested with *Not*I and cloned into the pCAGGS-MCS expression vector.

Table S1.

Specific oligonucleotide primers used to amplify anti-CEA-011 and 022 scFv

#1: 5’-CGAATgcggccgcGCCACCATGGGTTGGAGCTGTATCATCTTCTTTC-3’
#2: 5’-CAGAACCACCACCCCCGGCTGAGGAGACGGTGACTGAGGTTC-3’
#3: 5’-CGAATgcggccgcGCCACCATGCAGGTCCAGCTGCAGCAGTCTGGGGCTCAG-3’
#4: 5’-GGTGGAGGAGGTTCTGACATCCAGATGACTCAGTCTCCAGC-3’
#5: 5’-CGAATgcggccgcCCCGTCTGATTTCTAGCTTGGTGCC-3’
#6: 5’-GGTGGAGGAGGTTCTGACATCCTGATGACTCAGTCTCCAGC-3’
#7: 5’-CGAATgcggccgcCCGGTTTGATTTCCAGCTTGGTGCC-3’
#8: 5’-TCTGGATGTCAGAACCTCCTCCACCTGATCCTCCACCTCCAGAACCACCACCCCC-3’
#9: 5’-TCAGGATGTCAGAACCTCCTCCACCTGATCCTCCACCTCCAGAACCACCACCCCC-3’
#10: 5’-CTCCTCAGCCGGGGGTGGTGGTTCTGGAGGTGGAGGATCAGGTGGAGGAGGTTCT-3’
#11: 5’-CTCCGggatccGCCACCATGCAGGTCCAGCTGCAG-3’
#12: 5’-CCGgtcgacCTACAGGTCCTCCTCGCTGATCAG-3’

Lowercase letters indicate restriction site for *Not*I (gcggccgc), *Bam*HI (ggatcc), and *Sal*I (gtcgac).

Supplementary information Figure S2. (Full-length gels/blots)

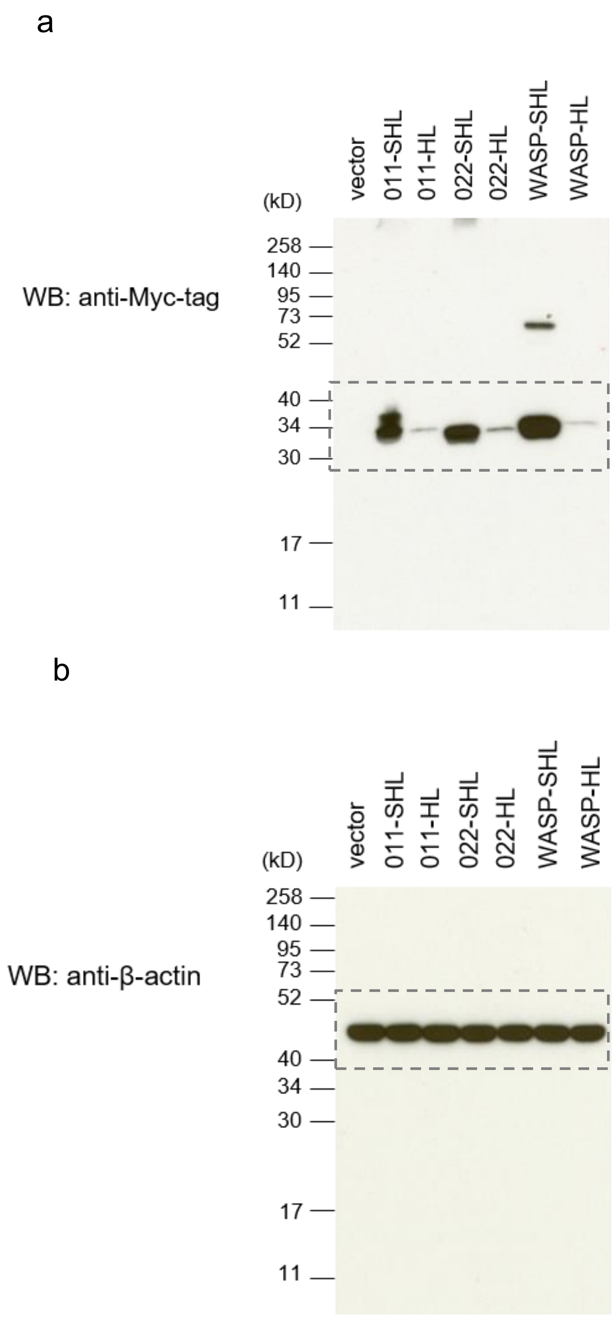

Figure S2. Western blot of anti-CEA 011-SHL, 011-HL, 022-SHL, 022-HL, anti-WASP-SHL, and-HL scFvs in transfected DO11-10 T cells. The Immunoblots were probed with (a) anti-Myc-tag polyclonal antibody or (b) anti-β-actin MAb. Each dotted line area indicates cropped blots in the main figures.

Supplementary information Figure S3

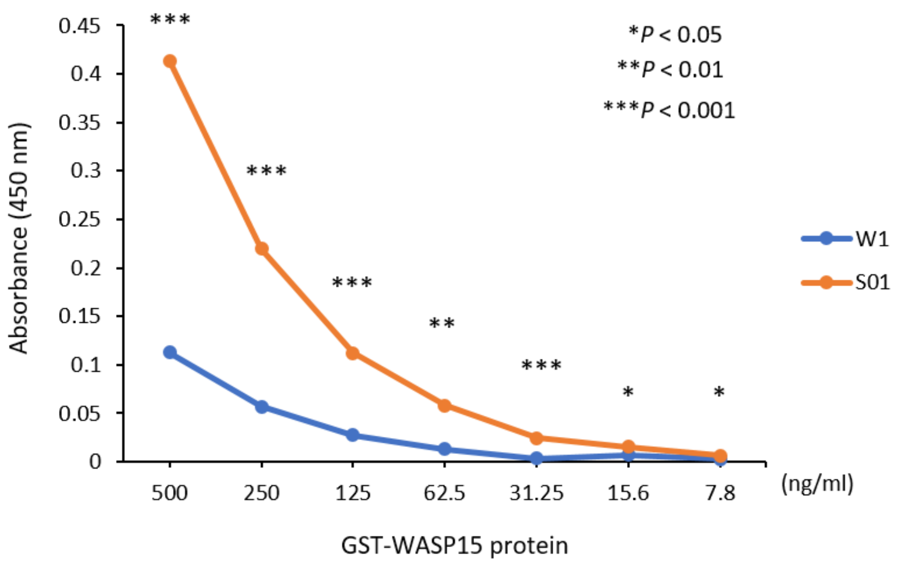

Figure S3. The specific binding activity of anti-WASP-scFv-conjugated FibL can be stored stably in the transgenic cocoon shells for nine years of storage at room temperature.

To investigate whether the cocoon shells can be stored at room temperature for many years without significant loss of their biological activities of scFv-conjugated FibL, S01 (FibL-anti-WASP-scFv) transgenic cocoon shells stored for nine years at room temperature and W1 (wild-type) cocoon shells were dissolved in aqueous LiBr solution, diluted with 1 mM Tris-HCl (pH 8.0) to a concentration of 0.125 mg/mL, and then applied to 96-well plates and incubated overnight at 4°C. After three washes with PBS, each well was blocked with ELISA Assay Diluent (BioLegend, San Diego, CA, USA) at room temperature for 1 h. After five washes with PBS and Tween 20, the indicated amount of GST-WASP15 fusion protein was applied to the wells and incubated at room temperature for 2 h. Binding was detected via sequential incubation of plates with anti-GST polyclonal antibody and HRP-conjugated anti-rabbit immunoglobulins, followed by incubation with ELISA POD Substrate TMB solution

(Nacalai Tesque). After colour development, the reaction was stopped with 2 N H_2SO_4 and the absorbance read at 450 nm using a microplate reader (iMark™ Microplate Reader; Bio-Rad). The specific binding activity of anti-WASP-scFv conjugated FibL prepared from "aged" cocoon shells was observed at similar levels to those from freshly obtained cocoon shells (Sato, M et al. *Sci Rep* 4, 4080 (2014)).
